# A key role of the PGC-1α/ERR-α pathway in regulation of angiogenic factors in proliferative diabetic retinopathy

**DOI:** 10.3389/fendo.2025.1615103

**Published:** 2025-07-17

**Authors:** Ahmed M. Abu El-Asrar, Mohd I. Nawaz, Ajmal Ahmad, Mairaj M. Siddiquei, Eef Allegaert, Priscilla W. Gikandi, Gert De Hertogh, Ghislain Opdenakker

**Affiliations:** ^1^ Department of Ophthalmology, College of Medicine, King Saud University, Riyadh, Saudi Arabia; ^2^ Dr. Nasser Al-Rashid Research Chair in Ophthalmology, College of Medicine, King Saud University, Riyadh, Saudi Arabia; ^3^ Laboratory of Histochemistry and Cytochemistry, University of Leuven, Leuven, Belgium; ^4^ University Hospitals UZ Gasthuisberg, Leuven, Belgium; ^5^ Laboratory of Immunobiology, Department of Microbiology, Immunology and Transplantation, Rega Institute, University of Leuven, Leuven, Belgium

**Keywords:** proliferative diabetic retinopathy, angiogenesis, PGC1-α, ERR-α, VEGF

## Abstract

**Background:**

PGC-1α is induced by hypoxia and interacts with the receptor ERR-α to stimulate angiogenic factors expression and promote angiogenesis. We investigated the possible role of the PGC-1α/ERR-α pathway in regulating angiogenic factors expression in proliferative diabetic retinopathy (PDR).

**Methods:**

We analysed vitreous fluid samples from PDR and non-diabetic patients and epiretinal fibrovascular membranes from PDR patients. Streptozotocin-treated rats were used as a model of diabetic retinopathy. Vitreous samples, epiretinal membranes, rat retinas, human retinal Müller glial cells and human retinal microvascular endothelial cells (HRMECs) were studied by Western blot analysis, ELISA and immunohistochemistry. Levels of reactive oxygen species (ROS) were determined with spectrofluorometric analysis.

**Results:**

Immunohistochemical analysis demonstrated co-expression of PGC-1α and ERR-α in endothelial cells and leukocytes in epiretinal membranes. Angiogenic activity, determined by the numbers of CD31-positive vessels, correlated significantly with PGC-1α and ERR-α expression levels. PGC-1α, ERR-α and the angiogenic biomarkers vascular endothelial growth (VEGF) and angiopoietin 2 were significantly increased in PDR vitreous samples. Diabetes induced upregulation of PGC-1α and ERR-α immunoreactive proteoforms in rat retinas. Cultured Müller cells and HRMECs constitutively expressed PGC-1α and ERR-α. In Müller cells, the PGC-1α inhibitor SR-18292 and the ERR-α selective inverse agonist XCT790 significantly attenuated VEGF, angiopoietin 2 and MCP-1/CCL2 upregulation induced by diabetic mimetic conditions. Treatment of Müller cells with the PGC-1α activator XLN005 induced significant upregulation of VEGF and attenuated ROS production induced by diabetic mimetic conditions.

**Conclusions:**

Our findings suggest that suppression of the PGC-1α/ERR-α pathway might impair the upregulation of angiogenic factors in PDR.

## 1 Introduction

Ischemia-induced retinal angiogenesis, the formation of new blood vessels from pre-existing vessels, plays a critical role in the initiation and progression of proliferative diabetic retinopathy (PDR). Retinal neovascularization and expansion of the extracellular matrix resulting in the outgrowth of fibrovascular membranes at the vitreoretinal interface often leads to serious vision loss due to recurrent vitreous hemorrhage and/or traction retinal detachment. Angiogenesis is tightly controlled by a balance of pro-angiogenic and anti-angiogenic factors (1). Vascular endothelial growth factor (VEGF), released in response to hypoxia, is a key driver of retinal vascular leakage and angiogenesis in the ocular microenvironment of patients with PDR and is the best-characterized proangiogenic factor in PDR (2). VEGF exerts its angiogenic effects by binding to its transmembrane tyrosine kinase receptor VEGF-R2 which is expressed on vascular endothelial cells and is the major receptor for pathological angiogenesis as well as microvascular permeability (3). Targeting the VEGF/VEGF-R2 pathway has emerged as an important therapeutic approach to arrest progression of angiogenesis in patients with PDR (4). However, these approaches often lead to transient responses as angiogenesis is regulated by multiple pathways and when the activity of one pathway, such as VEGF/VEGF-R2 is suppressed, the expression of other compensatory angiogenic pathways may appear and contribute to limit the efficacy of anti-VEGF treatment (5). Therefore, understanding the pathophysiology of PDR and identifying other pathways that regulate angiogenic factors and angiogenesis in the ocular microenvironment is of great interest.

In PDR, hypoxia seems to be a critical stimulator for neovascularization by upregulating the production of angiogenic factors (2, 6). Hypoxia-mediated induction of VEGF has been demonstrated in retinal cells (7, 8). During hypoxia, the oxygen-sensitive-hypoxia-inducible factor-1α (HIF-1α) accumulates in cells and heterodimerizes with the constitutively expressed HIF-1ß subunit, triggering the activation of many genes encoding proteins that regulate angiogenesis, such as VEGF (9, 10). The transcriptional coactivator PGC-1α (peroxisome proliferator-activated receptor-γ coactivator-1α) is induced by hypoxia and interacts with the orphan nuclear receptor estrogen-related receptor-α (ERR-α) to stimulate VEGF, angiopoietin 2 and other angiogenic factors expression and promote angiogenesis in cultured muscle cells and skeletal muscle *in vivo*, as well as in cancer cells (11). The induction of VEGF by the PGC-1α/ERR-α axis is independent of the HIF-1α pathway (11–13). PGC-1α is also a major regulator of mitochondrial function and oxidative metabolism in numerous tissues (14, 15). Therefore, the present study was designed to investigate whether and how the PGC-1α/ERR-α pathway was involved in angiogenic factors expression in the ocular microenvironment of patients with PDR, which is, unlike cancer, a genetically stable pathology.

## 2 Methods

### 2.1 Human retinal Müller glial cell and human retinal microvascular endothelial cell cultures

Human retinal Müller glial cells (MIO-M1) (a generous gift from Prof. A. Limb, Institute of Ophthalmology, University College London, UK) were cultured in Dulbecco’s Minimal Essential Medium (DMEM) containing 1 g/L glucose with 10% (v/v) fetal bovine serum and 100 units/mL of Penicillin-Streptomycin solution. Confluent cells were starved overnight in serum-free DMEM to minimize the effects of serum. Subsequently, the cell cultures were either left untreated or stimulated for 24 h.

Human retinal microvascular endothelial cells (HRMECs) were purchased from Cell Systems Corporation (Kirkland, WA, USA) and maintained in complete serum-free media (Cat No SF-4Z0–500, Cell System Corporation) supplemented with “Rocket Fuel” (Cat No SF-4Z0-500, Cell System Corporation), “Culture Boost” (Cat No 4CB-500, Cell System Corporation) and antibiotics (Cat No 4Z0–643, Cell System Corporation) at 37°C in a humidified atmosphere with 5% CO_2_. We used HRMECs up to passage 8 for all of our experiments. Cell cultures at about 80% confluency were starved in a minimal medium (medium supplemented with 0.25% “Rocket Fuel” and antibiotics) overnight to eliminate any residual effects of growth factors.

The following stimuli were used: diabetic mimetic conditions included treatment of Müller cells or HRMECs with 300 μM of the hypoxia mimetic agent cobalt chloride (CoCl_2_) (Cat No A1425-L, Avonchem Limited, UK), or 25mM glucose (Cat No GL0125100, Scharlau S.L, Gato Prez, Spain). For high-glucose (HG) treatment, 25 mM mannitol (Cat No MA01490500, Scharlau S.L, Gato Prez, Spain) was used as a control.

Müller cells were treated with 10 μM of the ERR-α inhibitor XCT790 (Cat No X4752, Sigma) or 20 μM of the PGC-1α inhibitor SR-18292 (Cat No SML2146, Sigma) for 24 h to determine their inhibitory effect. Müller cells were treated with 20 μM of the PGC-1α inducer ZLN005 (Cat No SML0802, Sigma) for 48 h.

Next, the treatment of human Müller cells with diabetic mimetic conditions, 300 μM of the hypoxia mimetic agent CoCl_2_ or 25mM glucose for 24 h was performed in the absence or presence of 1 h pretreatment with 10 μM of the ERR-α inhibitor XCT790, or 20 μM of the PGC-1α inhibitor SR-18292, or 10 μM of the HIF-1 α inhibitor YC-1 (Cat No Y102, Sigma).

After the treatment as detailed above, cell supernatants were collected and processed for enzyme-linked immunosorbent assay (ELISA) analysis. Harvested cells were lysed in a radioimmunoprecipitation assay (RIPA) lysis buffer (sc-24948, Santa Cruz Biotechnology, Inc.) for Western blot analysis.

### 2.2 Induction of streptozotocin-induced diabetes in rats

As described previously (16, 17), adult male Wistar rats of 8–9 weeks of age (200–220 g) were fasted overnight, and a single-bolus dose of streptozotocin of 60 mg/kg in 10 mM sodium citrate buffer, pH 4.5, (Sigma, St. Louis, MO, USA) was injected intraperitoneally. Equal volumes of citrate buffer were injected in age-matched control rats. Rats were considered diabetic if their blood glucose levels were in excess of 250 mg/dL. The rat model of diabetic retinopathy demonstrates the early retinal changes of nonproliferative diabetic retinopathy that occur in humans, such as inflammation and breakdown of the blood-retinal barrier resulting in increased vascular permeability (18, 19). After 4 weeks of diabetes, the rats were euthanized, and retinas were isolated and frozen immediately in liquid nitrogen and stored at -80°C until analyzed. Similarly, retinas were obtained from age-matched nondiabetic control rats.

### 2.3 Vitreous samples and epiretinal membranes specimens

Undiluted vitreous fluid samples (0.3–0.6 ml) were obtained from 37 patients with PDR during pars plana vitrectomy, for the treatment of tractional retinal detachment, and/or nonclearing vitreous hemorrhage and processed as described previously (16, 17). We compared the samples from diabetic patients with those of a clinical control cohort. The control group consisted of 30 patients who had undergone vitrectomy for the treatment of rhegmatogenous retinal detachment with no proliferative vitreoretinopathy. Control subjects were clinically checked to be free from diabetes or other systemic disease. Epiretinal fibrovascular membranes were obtained from 13 patients with PDR during pars plana vitrectomy for the repair of tractional retinal detachment. The epiretinal membranes were processed as previously described (16, 17). Membranes were fixed for 2h in 10% formalin solution and embedded in paraffin. Patients with PDR were 25 (67.6%) males and 12 (32.4%) females, whose ages ranged from 35 to 75 years with a mean of 54.1 ± 10.8 years. The duration of diabetes ranged from 8 to 35 years with a mean of 19.3 ± 7.4 years. Twenty-two patients had insulin-dependent diabetes mellitus, and 15 patients had noninsulin-dependent diabetes mellitus. Treatment for hypertension was used by 18 patients, 6 patients had diabetic nephropathy, and 2 patients had cardiovascular disease. The control group included 20 (66.7%) males and 10 (33.3%) females, whose ages ranged from 29 to 75 years with a mean of 53.3 ± 15.9 years. There were no significant differences in the male to female ratio (p=0.938) and age (p=0.820) between patients with PDR and control patients.

### 2.4 Enzyme-linked immunosorbent assays

ELISA kits for human vascular endothelial growth factor (VEGF) (Cat No DY293B), human monocyte chemoattractant protein-1 (MCP-1)/CCL2 (Cat No DY279), human angiopoietin 2 (Cat No DY623), and human matrix metalloproteinase-9 (MMP-9) (Cat No DY911) were purchased from R&D Systems, Minneapolis, MA, USA. An ELISA kit for human PGC-1α (Cat No MBS772836) was purchased from MY Biosource (San Diego, USA).

Levels of VEGF, angiopoietin 2, and PGC-1α in vitreous fluid; and VEGF, MCP-1, angiopoietin 2, and MMP-9 in cell culture medium were determined using the afore-mentioned ELISA kits according to the manufacturer’s instructions. The minimum detection limits for VEGF, MCP-1, angiopoietin-2, MMP-9, and PGC-1α ELISA kits were approximately 12 pg/ml, 9 pg/ml, 10 pg/ml, 10 pg/ml, 10 pg/ml, respectively.

### 2.5 Western blot analysis of human vitreous fluid, human retinal Müller glial cells and human retinal microvascular endothelial cell lysates, and rat retinas

Retina and cell lysates were homogenized in Western blot lysis buffer [30 mM Tris-HCl; pH 7.5, 5 mM EDTA, 1% Triton X-100, 250 mM sucrose, 1 mM sodium vanadate, and a complete protease inhibitor cocktail from Roche (Mannheim, Germany)]. After centrifugation of the homogenates (14,000 X g for 15 min, 4°C), protein concentrations were measured in the supernatants (Bradford protein assay kit; Bio-Rad Laboratories, Hercules, CA, USA). Equal amounts, either 30 µg or 50 µg of the protein extracts from lysates were subjected to SDS–PAGE and transferred onto nitrocellulose membranes.

To determine the presence of PGC-1α and ERR-α in the vitreous fluid samples, equal volumes (10 μL) of vitreous samples were boiled in Laemmli’s sample buffer (1:1, v/v) under reducing condition for 10 min.

Immunodetection was performed with the use of rabbit polyclonal anti-PGC-1α antibody (1:1000, ab54481, Abcam), mouse monoclonal anti-PGC-1α antibody (1:1000, MAB10784-SP, R&D Systems), rabbit polyclonal anti-PGC-1α antibody (1:1000, NBP104676, Novus Biologicals, LLC, Centennial, USA), mouse monoclonal anti-ERR-α antibody (1:1000, sc-65718, Santa Cruz Biotechnology Inc., Santa Cruz, CA, USA), rabbit polyclonal anti-ERR-α (1:1000, Cat. no. ab137489, Abcam).

Nonspecific binding sites on the nitrocellulose membranes were blocked (1.5 h, room temperature) with 5% non-fat milk made in Tris-buffered saline containing 0.1% Tween-20 (TBS-T). Three TBS-T washings (5 min each) were performed before the secondary antibody treatment at room temperature for 1 h. The secondary antibodies included goat anti-rabbit immunoglobulin (SC-2004) and goat anti-mouse immuno-globulin (SC-2005) (1:2000, Santa Cruz Biotechnology Inc.). To verify equal loading, the nitrocellulose membranes were stripped and reprobed either with β-actin-specific antibody (1:2000, sc-47778, Santa Cruz Biotechnology Inc.) or β-tubulin-specific antibody (1:2000, ab21058, Abcam). Bands were visualized with the use of high-performance chemiluminescence (G: Box Chemi-XX9 from Syngene, Synoptic Ltd., Cambridge, UK), and the band intensities were quantified with the use of GeneTools software (Syngene by Synoptic Ltd.).

### 2.6 Reactive oxygen species assay

Reactive oxygen species (ROS) generation was measured in Müller glial cell monolayers using 2′-7′-dichlorofluorescin-diacetate (DCFH-DA). Briefly, cells were grown in standard cell culture media so that 3 x 10^6^–4 x 10^6^ cells were obtained the day before the experiment. Next, cells were harvested and seeded in a clear bottom 24-well microplate with 1 x 10^5^ cells per well. Cells were allowed to adhere overnight. Overnight starved cells were treated either with 10 mM hydrogen peroxide (H_2_O_2_) for 1 h or 25mM glucose for 24 h in the absence or presence of 48 h pretreatment with the PGC-1α inducer ZLN005 (20 μM). For high-glucose (HG) treatment, 25 mM mannitol was used as a control. After washing with 250 μL/well of PBS, the cells were stained by adding 200 μL/well of the 10 μM DCFH-DA (Invitrogen, CA) for 45 minutes at 37°C in the dark. After removing DCFH-DA solution, cell monolayers were washed with PBS and Cellular ROS production was measured immediately on a fluorescence plate reader (SpectraMax Gemini-XPS, Molecular Devices, CA, USA) with excitation and emission wavelengths of 488 nm and 525 nm, respectively. To normalize the fluorescence intensities with protein concentrations, cells were lysed and 1 μL of the supernatant transferred to a clear 96 well plate containing 100 μL of 1:5 diluted protein assay (Bradford assay) solution to measure the protein concentration.

### 2.7 Immunohistochemical staining for epiretinal membranes

The immunohistochemical and the sequential double immunohistochemical stainings were performed using the Leica Bond Max autostainer system (M496834 -Leica, Diegem, Belgium) with Bond Polymer Refine Red Detection kit (DS9390, Leica) and Bond Polymer Refine Detection kit (DS9800, Leica). The Bond Polymer Refine Red Detection kit is a biotin-free, polymeric alkaline phosphatase (AP)-linker antibody conjugate system for the detection of tissue-bound mouse and rabbit IgG and some mouse IgM primary antibodies. Bond Polymer Refine Red Detection utilizes a novel controlled polymerization technology to prepare polymeric AP-linker antibody conjugates. The Bond Polymer Refine Detection is a biotin-free, polymeric horseradish peroxidase (HRP)-linker antibody conjugate system for the detection of tissue-bound mouse and rabbit IgG and some mouse IgM primary antibodies. Bond Polymer Refine Detection utilizes a novel controlled polymerization technology to prepare polymeric HRP-linker antibody conjugates.

The Bond Polymer Refine Red Detection works as follows:

- A user-supplied specific primary antibody is applied.
- Post Primary IgG linker reagent localizes mouse antibodies.
- Poly-AP IgG reagent localizes rabbit antibodies.
- The substrate chromogen, Fast Red, visualizes the complex via a red precipitate.
- Hematoxylin (blue) counterstaining allows the visualization of cell nuclei.

The Bond Polymer Refine Detection works as follows:

- A user-supplied specific primary antibody is applied.
- Post Primary IgG linker reagent localizes mouse antibodies.
- Poly-HRP IgG reagent localizes rabbit antibodies.
- The substrate chromogen, 3,3’-Diaminobenzidine tetrahydrochloride hydrate (DAB), visualizes the complex via a brown precipitate.
- Hematoxylin (blue) counterstaining allows the visualization of cell nuclei.

Sections were cut at 3-µm thick with a microtome (HistoCore MULTICUT-Semi-Automated Rotary Microtome; reference: 14051856372 - Leica). The used slides are from Dako (IHC Microscope slides – reference: K8020). We examined 75 slides. The paraffin-embedded tissue sections, the primary antibodies and the Bond Polymer Refine Red Detection kit (DS9390, Leica) were loaded onto the Bond Max autostainer and dewaxed (AR9222, Leica), followed by antigen retrieval. For CD31 and ERR-α, antigen retrieval was performed by boiling (99°C) the sections in citrate based buffer [pH 5.9–6.1] [ER1-AR9961, Bond Epitope Retrieval Solution 1; Leica] for 20 minutes. For CD45 and PGC-1α detection, antigen retrieval was performed by boiling (99°C) the sections in Tris/EDTA buffer [pH 9] [ER2-AR9640, Bond Epitope Retrieval Solution 2; Leica] for 20 minutes. Subsequently, the sections were incubated for 60 minutes with mouse monoclonal anti-CD31 (ready-to-use; clone JC70A; Dako, Glostrup, Denmark), mouse monoclonal anti-CD45 (ready-to-use; clones 2B11+PD7/26; Dako), rabbit polyclonal anti-PGC-1α antibody (1:100; ab54481, Abcam, Cambridge, UK) and rabbit polyclonal anti-ERR-α antibody (1:500; ab137489, Abcam). Optimal working conditions for the antibodies were determined in pilot experiments on kidney and heart sections. Then the sections were incubated with secondary antibodies, first with a post primary IgG for 20 mins, followed by a poly-AP IgG for 30 mins. The reaction product was visualized by incubation for 2 times 15 minutes with the Fast Red chromogen, resulting in bright-red immunoreactive sites. The slides were then faintly counterstained with hematoxylin for 5 mins. In between each step sections were washed with wash buffer (AR 9590, Leica).

To identify the phenotype of cells expressing PGC-1α and ERR-α, sequential double immunohistochemistry was performed. A Bond Polymer Refine Red Detection kit (DS9390, Leica) was used in combination with a Bond Polymer Refine Detection kit (DS9800, Leica). After dewaxing and antigen retrieval, the sections were incubated for 60 mins with the first primary antibody (anti-CD45) and subsequently treated with peroxidase-conjugated secondary antibody (post primary IgG linker reagent) for 8 mins to define the leukocytes. The resulting immune complexes were visualized by enzymatic reaction of the 3, 3’-diaminobenzidine tetrahydrochloride substrate for 5 mins. Incubation of the second primary antibodies (anti-PGC-1α and ERR-α) for 60 mins was followed by treatment with alkaline phosphatase-conjugated secondary antibody (poly-HRP IgG reagent) for 30 mins and fast red reactions for 2 times 15 mins. No counterstain was applied. Negative controls were by omission of the primary antibody from the staining protocol. Instead, the ready-to-use DAKO Real antibody Diluent (Agilent Technologies Product Code 52022) was applied.

### 2.8 Quantitation

The level of vascularization in epiretinal fibrovascular membranes was determined by immunodetection of the vascular endothelium marker CD31. Immunoreactive blood vessels and cells were counted in five representative fields, with the use of an eyepiece calibrated grid in combination with the 40x objectives. These representative fields were selected based on the presence of immunoreactive blood vessels and cells. With this magnification and calibration, immunoreactive blood vessels and cells present in an area of 0.33 mm × 0.22 mm were counted.

### 2.9 Statistical analysis

Data were collected, stored and managed in a spreadsheet using Microsoft Excel 2010^®^ software. Data were analyzed and figures prepared using SPSS^®^ version 21.0 (IBM Inc., Chicago, Illinois, USA). Age and gender of the human subjects from whom the samples were obtained were matched for cases and controls; independent t-test for age and Chi-square test for gender were used to test the differences between the two groups. Tests for normality were done using Shapiro-Wilk test and Q-Q plots. The data were normally distributed and hence reported as mean and standard deviation (SD) and illustrated with bar charts. Consequently, One-Way ANOVA and Independent t-tests (applying Bonferroni correction where necessary) were done to test the differences between the groups. Additionally, Pearson’s correlation analysis was carried out. Any output with a p-value below 0.05 was interpreted as an indicator of statistical significance.

## 3 Results

### 3.1 Expression of PGC-1α and ERR-α in epiretinal fibrovascular membranes from patients with PDR

**Figure 1:**
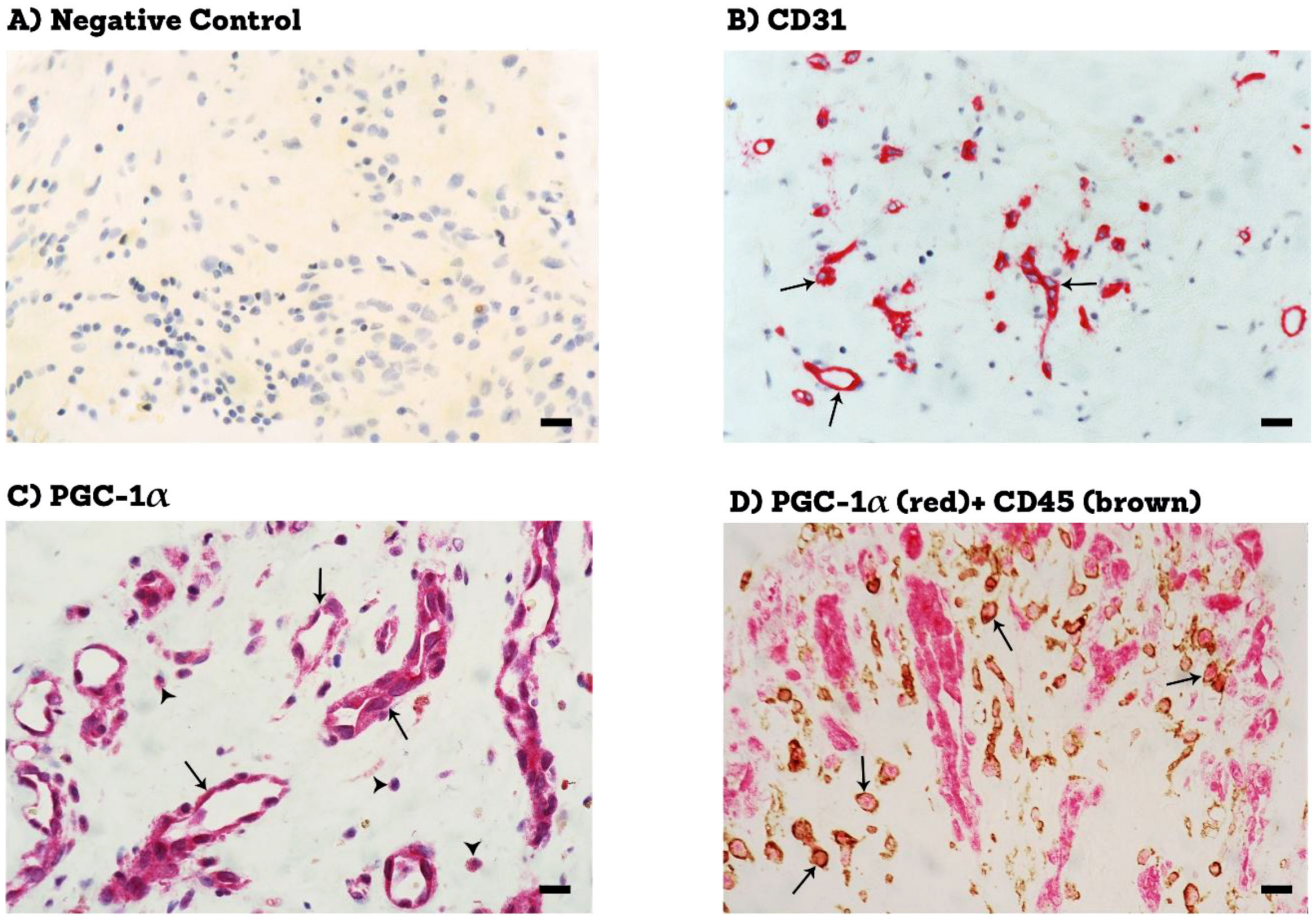
Immunohistochemical staining of proliferative diabetic retinopathy epiretinal fibrovascular membranes. **(A)** negative control slide (procedure without the addition of the primary antibody) showing no labelling. **(B)** staining for the endothelial cell marker (CD31 showing pathologic new blood vessels (arrows). **(C)** staining for PGC-1α showing immunoreactivity in vascular endothelial cells (arrows) and in stromal cells (arrowheads). **(D)** double staining for PGC-1α (red) and CD45 (brown) showing co-expression in stromal cells. No counterstain to visualize the cell nuclei was applied (arrows) (black scale bar, 10 µM).

**Figure 2:**
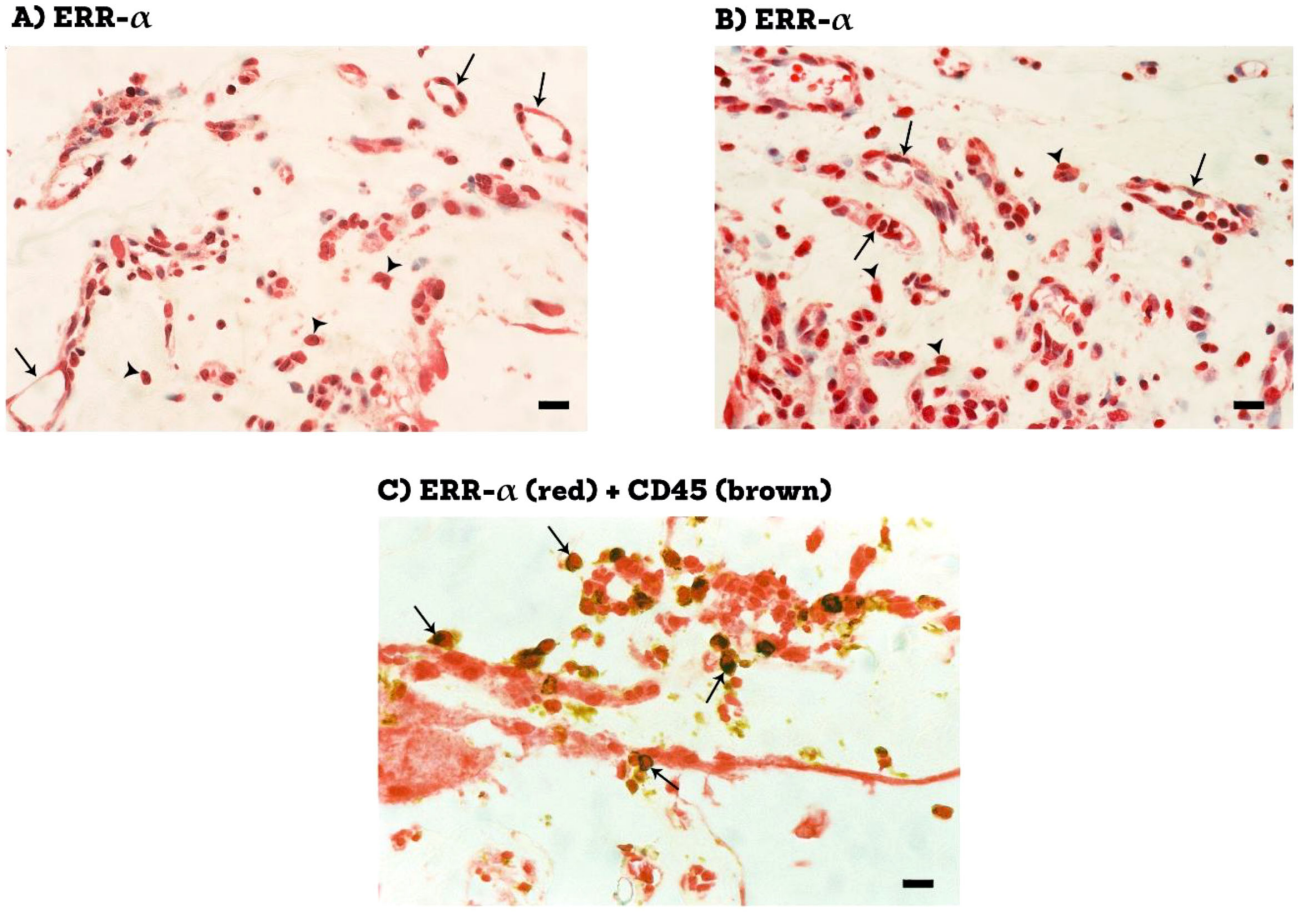
Immunohistochemical staining of proliferative diabetic retinopathy epiretinal fibrovascular membranes. Staining for ERR-α showing immunoreactivity in vascular endothelial cells (arrows) and in stromal cells (arrowheads) **(A, B)**. Double staining for ERR-α (red) and CD45 (brown) showing co-expression in stromal cells. No counterstain to visualize the cell nuclei was applied (arrows) **(C)** (black scale bar, 10 µM).

**Figure 3:**
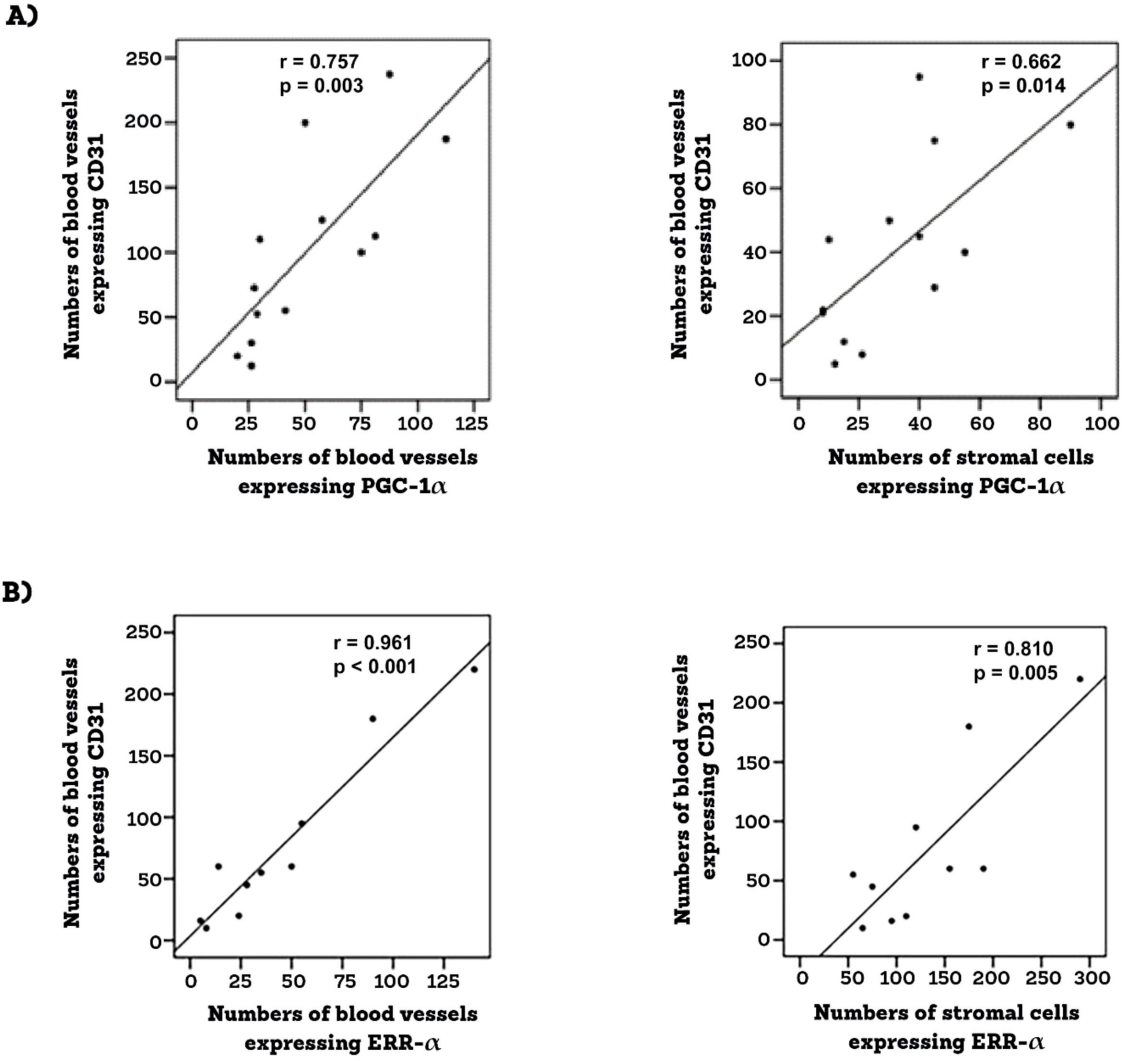
Epiretinal membranes from PDR patients were studied by immunohistochemical analysis. Significant positive correlations between angiogenic activity (CD-31-positive vessels) and the expressions of PGC-1 α **(A)** and ERR-α **(B)** (Pearson’s correlation coefficient).

Epiretinal fibrovascular membranes were studied by immunohistochemical analysis to examine the expression and tissue localization of PGC-1α (n=13) and ERR-α (n=10). No staining was observed in the negative control slides (**Figure 1A**). All membranes showed pathologic neovessels expressing the vascular endothelial cell marker CD31 (**Figure 1B**). Immunoreactivity for PGC-1α was detected in all membranes in endothelial cells lining pathologic neovessels and stromal cells (**Figure 1C**). Co-localization studies revealed that immunoreactivity for PGC-1α was detected in stromal leukocytes expressing CD45 (**Figure 1D**). In a way similar to PGC-1α, immunoreactivity for ERR-α was detected in vascular endothelial cells and stromal cells (**Figures 2A, B**). In line with its expected subcellular distribution, ERR-α was often visualized within cellular nuclei. Stromal cells were leukocytes co-expressing CD45 (**Figure 2C**). Significant positive correlations (Pearson’s correlation coefficient) were detected between the numbers of pathologic neovessels expressing CD31, reflecting the angiogenic activity, and the numbers of blood vessels (r=0.757; p=0.003) and stromal cells (r=0.662; p=0.014) ex-pressing PGC-1α (**Figure 3A**). Similarly, significant positive correlations were detected between the numbers of neovessels expressing CD31 and the numbers of blood vessels (r=0.961; p<0.001) and stromal cells (r=0.810; p=0.005) expressing ERR-α (**Figure 3B**).

### 3.2 Western blot analysis of vitreous samples

**Figure 4:**
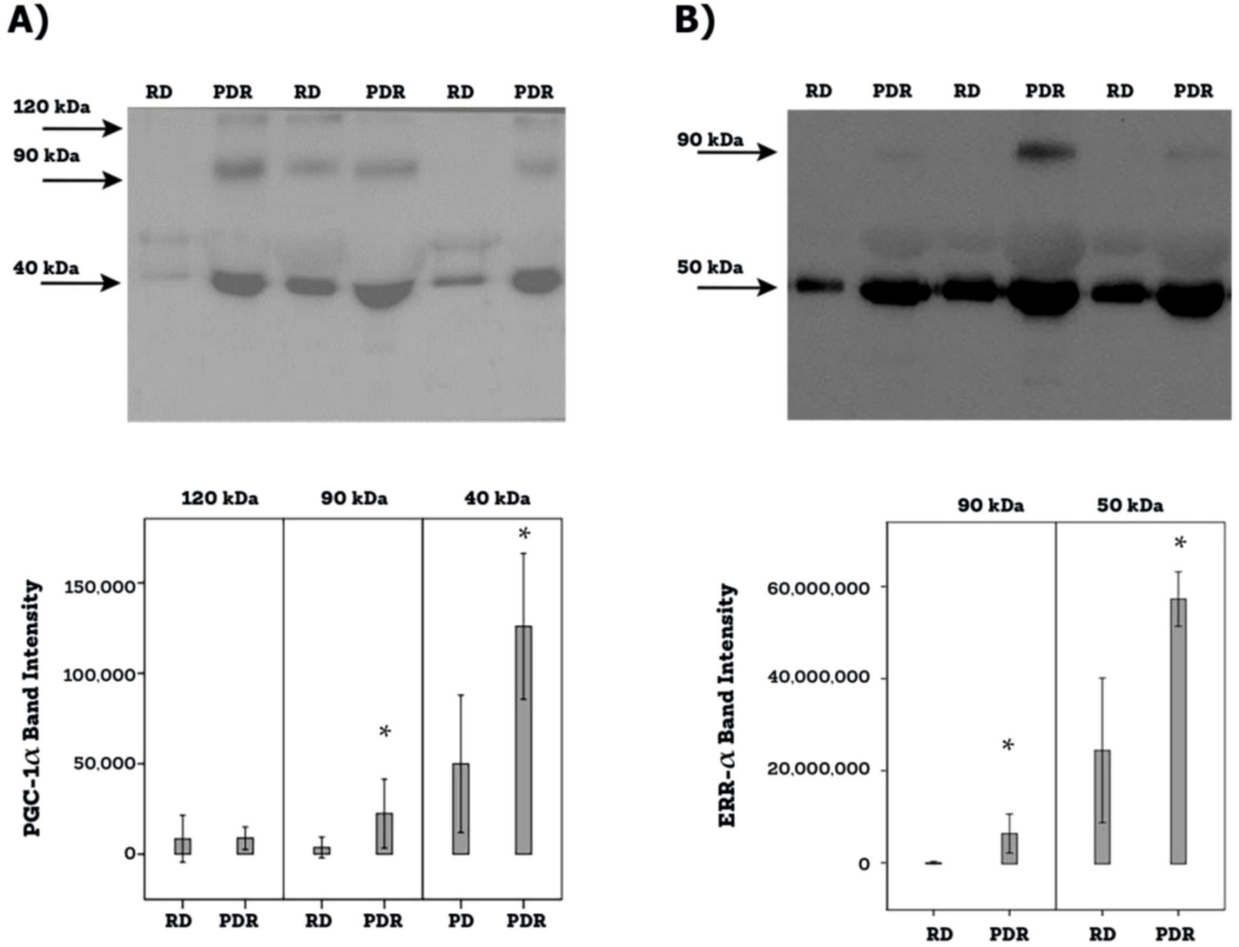
Determination of PGC-1α **(A)** and ERR-α **(B)** levels in vitreous fluid samples. Equal volumes (10 µl) of vitreous fluid from 12 patients with proliferative diabetic retinopathy (PDR) and from 12 non-diabetic patients with rhegmatogenous retinal detachment (RD) were subjected to gel electrophoresis and the presence of PGC-1α (Abcam antibody) and ERR-α were detected by Western blot analysis. Representative sets of samples are shown. The intensity of the protein bands was determined in all samples and band intensities were compared between RD and PDR patients. Results are expressed as mean ± standard deviation (p*<0.05; independent t-test).

Western blot analysis of equal volumes of vitreous fluid confirmed the presence of PGC-1α and ERR-α in the vitreous fluid. The presence of PGC-1α and ERR-α in the vitreous fluid reflects cell death accompanying the diabetic process as well as cell lysis induced by the freeze-thaw cycle. PGC-1α immunoreactivities were detected as three protein bands at approximately 120 kDa, 90 kDa and 40 kDa (**Figure 4A**). These corresponded to the full-length PGC-1α at about 90 kDa and 120 kDa. In all likelihood the predominant 40-kDa band represented the N-truncated PGC-1α isoform (20–23). This functional and biologically active proteoform of PGC-1α originates from alternative mRNA splicing introducing a premature stop codon (20–23). In **Figure 4A**, PGC-1α immunodetection was performed with the use of an antibody from Abcam. Additional control analyses with antibodies from R & D Systems and Novus biologicals, yielded similar immune reactivities. ERR-α immunoreactivities were expressed as 2 protein bands at approximately 90 kDa and 50 kDa (**Figure 4B**). Scanning analysis of immunoreactivities demonstrated increased levels of the 90-kDa and 40-kDa PGC-1α isoforms (**Figure 4A**) and both ERR-α forms (**Figure 4B**) in vitreous of PDR patients in comparison with controls.

### 3.3 ELISA levels of PGC-1α, VEGF and angiopoietin 2 in vitreous samples

To corroborate the above findings, we measured total immunoreactivities in vitreous samples. PGC-1α levels in vitreous samples from patients with PDR were significantly higher than the levels in nondiabetic controls (p<0.001) (**Table 1**). To allow correlation analysis with ongoing angiogenesis in the ocular microenvironment of patients with PDR, we analyzed the levels of the proangiogenic factors VEGF and angiopoietin 2. VEGF and angiopoietin 2 levels were significantly enhanced in vitreous samples from patients with PDR compared to nondiabetic control samples (p=0.005 and p<0.001, respectively) (**Table 1**). Significant positive correlations (Pearson’s correlation coefficient) were found between levels of PGC-1α and angiopoietin 2 (r=0.439; p<0.001) and between levels of VEGF and angiopoietin 2 (r=0.484; p<0.001).

**Table 1:** Comparisons of PGC-1α, vascular endothelial growth factor (VEGF) and angiopoietin 2 levels in vitreous samples from patients with proliferative diabetic retinopathy (PDR) and nondiabetic patients with rhegmatogenous retinal detachment (RD).

Variables	PDR (n=37) (Mean ± SD)	RD (n=30) (Mean ± SD)	p-value (Independent t-test)
· PGC-1α (pg/ml)	39.0 ± 44.5	8.1 ± 15.4	<0.001*
· VEGF (pg/ml)	698.5 ± 1003.3	130.9 ± 557.6	0.005 *
· Angiopoietin 2 (pg/ml)	3906.1 ± 3571.5	326.8 ± 540.3	<0.001*

^*^Statistically significant at 5% level of significance.

Next we performed subgroup analyses to investigate the effects of gender and type of diabetes on the levels of PGC-1α, VEGF and angiopoietin 2 in vitreous samples from patients with PDR. As shown in **Table 2**, there were no significant differences.

**Table 2:** Effects of gender and type of diabetes on the levels of PGC-1α, vascular endothelial growth factor (VEGF) and angiopoietin 2 in vitreous samples from patients with proliferative diabetic retinopathy.

Variables	Gender		p-value (Independent t-test)
	Male (n=25) (Mean ± SD)	Female (n=12) (Mean ± SD)	
· PGC-1α (pg/ml)	43.3 ± 42.7	27.3 ± 43.8	0.304
· VEGF (pg/ml)	634.7 ± 1052.3	684.3 ± 964.9	0.892
· Angiopoietin 2 (pg/ml)	3831.5 ± 3607.5	3941.2 ± 3929.4	0.934

Variables	Type of diabetes		p-value (Independent t-test)
	Insulin-dependent (n=22) (Mean ± SD)	Noninsulin-dependent (n=15) (Mean ± SD)	
· PGC-1α (pg/ml)	37.3 ± 44	38.6 ± 43.3	0.937
· VEGF (pg/ml)	718 ± 1177.5	524.6 ± 589.7	0.598
· Angiopoietin 2 (pg/ml)	3809.7 ± 3502.2	3982.8 ± 4114.1	0.897

### 3.4 Short-term effect of diabetes on retinal expression of PGC-1α and ERR-α in experimental rats

**Figure 5:**
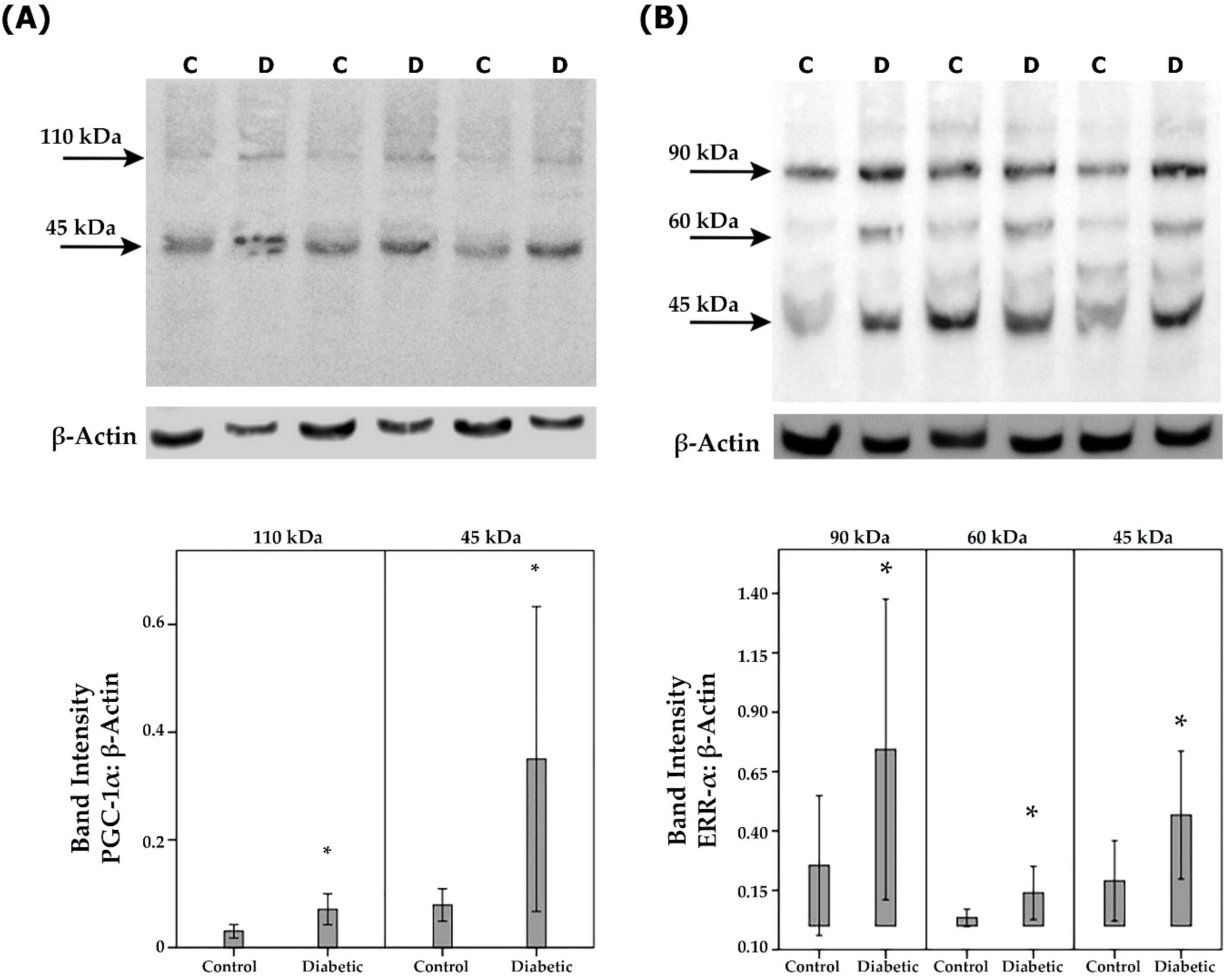
PGC-1α and ERR-α expression levels in diabetic rat retinas. PGC-1α **(A)** and ERR-α **(B)** expression in the retinal lysates of non-diabetic control (C) rats (n=12) and diabetic (D) rats (n=12) were determined by Western blot analysis. After determination of the intensity of the PGC-1α and ERR-α protein bands, intensities were adjusted to those of β-actin in the samples. Results are expressed as mean ± standard deviation (p*<0.05; independent t-test).

Next, we studied the regulation of retinal expression of PGC-1α and ERR-α in streptozotocin-induced diabetic rats. Whereas this animal model may not fully reflect all aspects of long-term diabetic retinopathy observed in the human samples illustrated above, it is accepted as a model to investigate short-term effects of acute inflammation associated with diabetic retinopathy. With the use of Western blot analysis of homogenized retinal tissue, we detected PGC-1α as 2 protein bands at approximately 110 kDa and 45 kDa. The predominant PGC-1α isoform was found at 45 kDa (**Figure 5A**). Densitometric analysis showed increased PGC-1α protein levels in the retina of rats after 4 weeks of STZ-induced diabetes (**Figure 5A**). ERR-α immunoreactivities were expressed as 3 protein bands at approximately 90 kDa, 60 kDa and 45 kDa (**Figure 5B**). Densitometric analysis demonstrated increased ERR-α protein levels in the retina of diabetic rats (**Figure 5B**).

### 3.5 Effect of diabetic retinopathy-associated mechanisms on the expression of PGC-1α and ERR-α in human retinal microvascular endothelial cells and human retinal Müller glial cells

**Figure 6:**
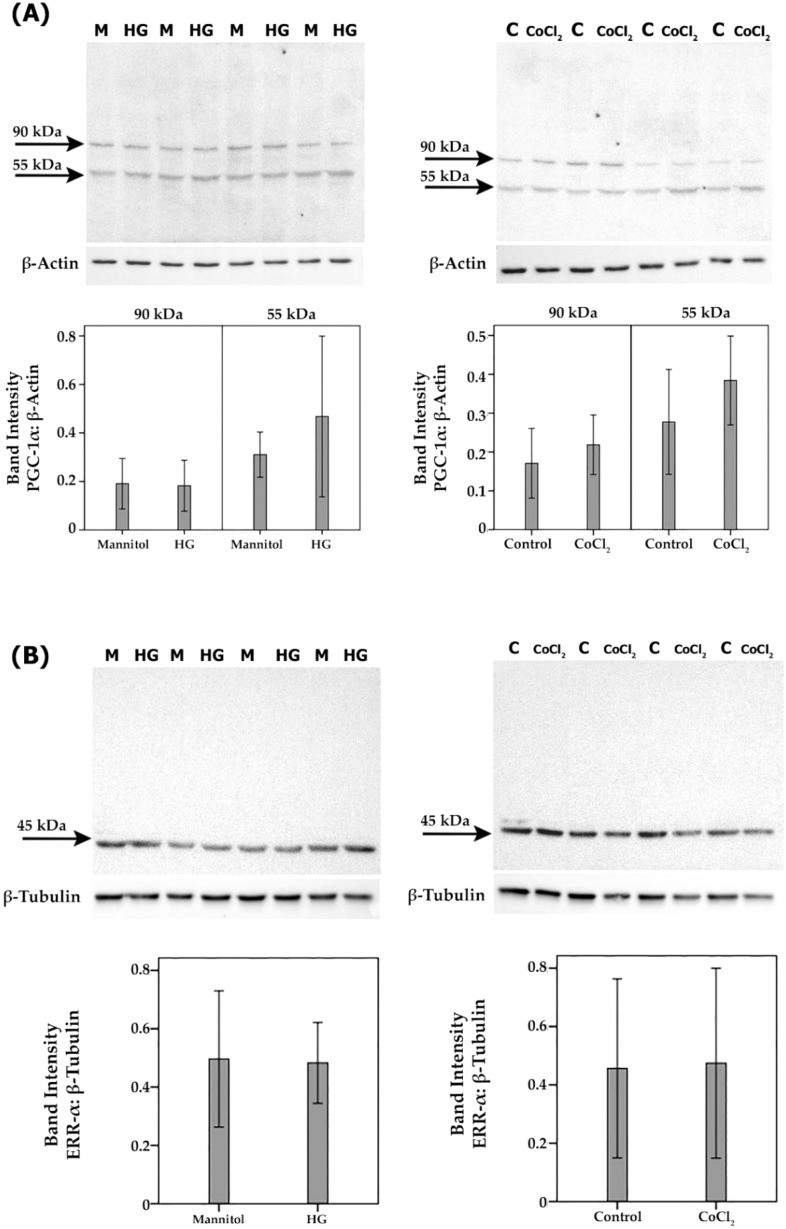
Human retinal microvascular endothelial cells (HRMECs) were left untreated as control (C), cultured in medium with 25mM mannitol (M) as control for high-glucose (HG) or treated with HG (25mM) or cobalt chloride (CoCl_2_) (300µM) for 24 h. Expression levels of PGC-1α **(A)** and ERR-α **(B)** in the cell lysates were determined by Western blot analysis. Results are expressed as mean ± standard deviation from three different experiments, each performed in triplicate.

**Figure 7:**
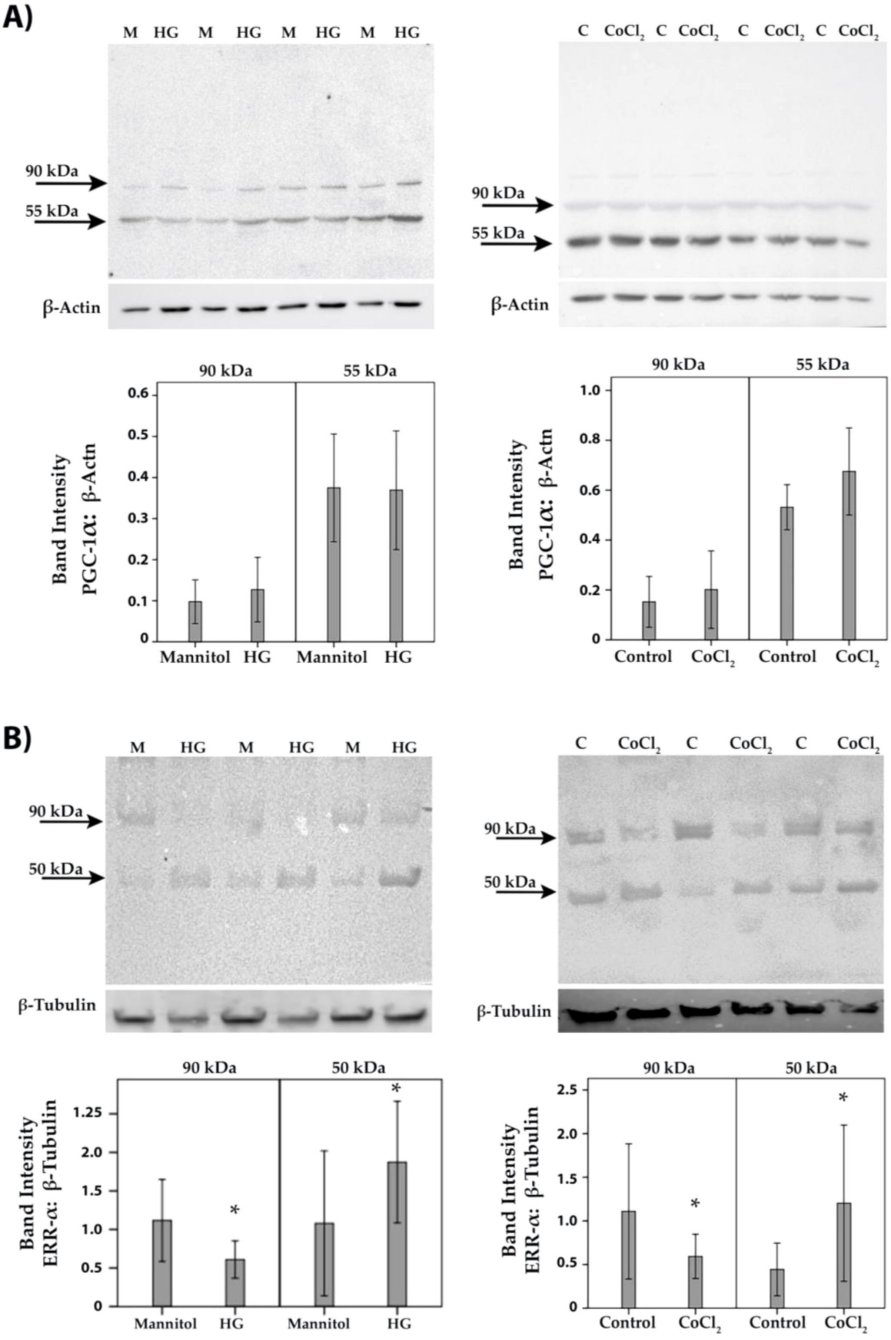
Human retinal Müller glial cells were left untreated (C) or treated with high-glucose (HG) (25mM) or cobalt chloride (CoCl_2_) (300 µM) for 24 h. Cell cultures containing 25 mM mannitol (M) were used as a control for HG. Expression levels of PGC-1α **(A)** and ERR-α **(B)** in the cell lysates were determined by Western blot analysis. Results are expressed as mean ± standard deviation from three different experiments, each performed in triplicate. (p* <0.05; independent t-test).

To confirm the observed *in vivo* expression of PGC-1α and ERR-α by endothelial cells in epiretinal fibrovascular membranes from patients with PDR, we performed *in vitro* experiments on HRMECs. We showed by Western blot analysis that HRMECs constitutively express PGC-1α and ERR-α (**Figure 6**). Treatment of HRMECs with high-glucose or the hypoxia-mimetic agent CoCl_2_ did not affect the expression of PGC-1α (**Figure 6A**) or ERR-α (**Figure 6B**). Similarly, Western blot analysis demonstrated that Müller cells constitutively express PGC-1α and ERR-α (**Figure 7**). Treatment of Müller cells with high-glucose or CoCl_2_ did not affect the expression of PGC-1α (**Figure 7A**). However, treatment with high-glucose or CoCl_2_ induced significant upregulation of the 50 kDa protein band of ERR-α and significant downregulation of the 90 kDa protein band of ERR-α (**Figure 7B**).

### 3.6 Effect of the PGC-1α inhibitor SR-18292 and the ERR-α inhibitor XCT790 on the expression of proangiogenic molecules in human retinal Müller glial cells

**Figure 8:**
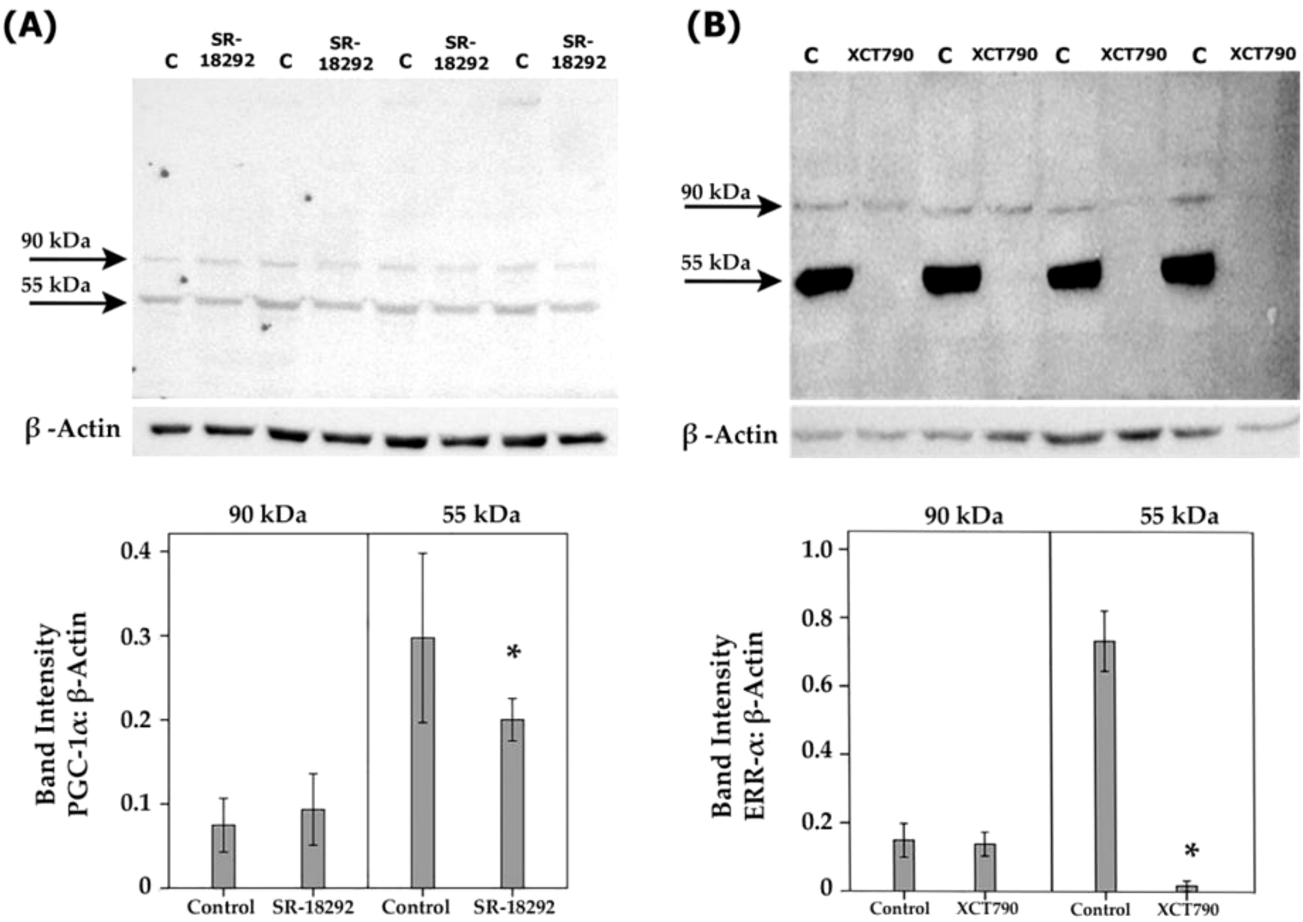
Human retinal Müller glial cells were left untreated or treated with SR-18292 (20 µM) for 24 h or XCT790 (10 µM) for 24 h. Expression levels of PGC-1 α **(A)** and ERR- α **(B)** in the cell lysates were determined by Western blot analysis. Results are expressed as mean ± standard deviation from three different experiments, each performed in triplicate. (*p <0.05; independent t-test).

By the demonstration that the PGC-1α/ERR-α axis is upregulated in the ocular microenvironment of patients with PDR and in the retina of diabetic rats, we started to dissect the effects of inhibiting the PGC-1α/ERR-α pathway on diabetic conditions at the cellular level *in vitro*. Müller cells were cultured in the absence or presence of SR-18292 or XCT790. With the use of Western blot analysis of cell lysates, we demonstrated that treatment with SR-18292 induced significant down-regulation of the 55 kDa protein band of PGC-1α (**Figure 8A**). Similarly, treatment of Müller cells with XCT790 induced significant downregulation of the protein levels of ERR-α (**Figure 8B**). With the use of ELISA analysis, we demonstrated that the treatment of Müller cells with SR-18292 or XCT790 did not affect the expression of the proangiogenic factors VEGF and angiopoietin 2 in the culture medium as compared to untreated control (data not shown).

### 3.7 Effect of the PGC-1α inhibitor SR-18292 and the ERR-α inhibitor XCT790 on high-glucose (HG)-induced upregulation of proangiogenic and inflammatory molecules in human retinal Müller glial cells

**Figure 9:**
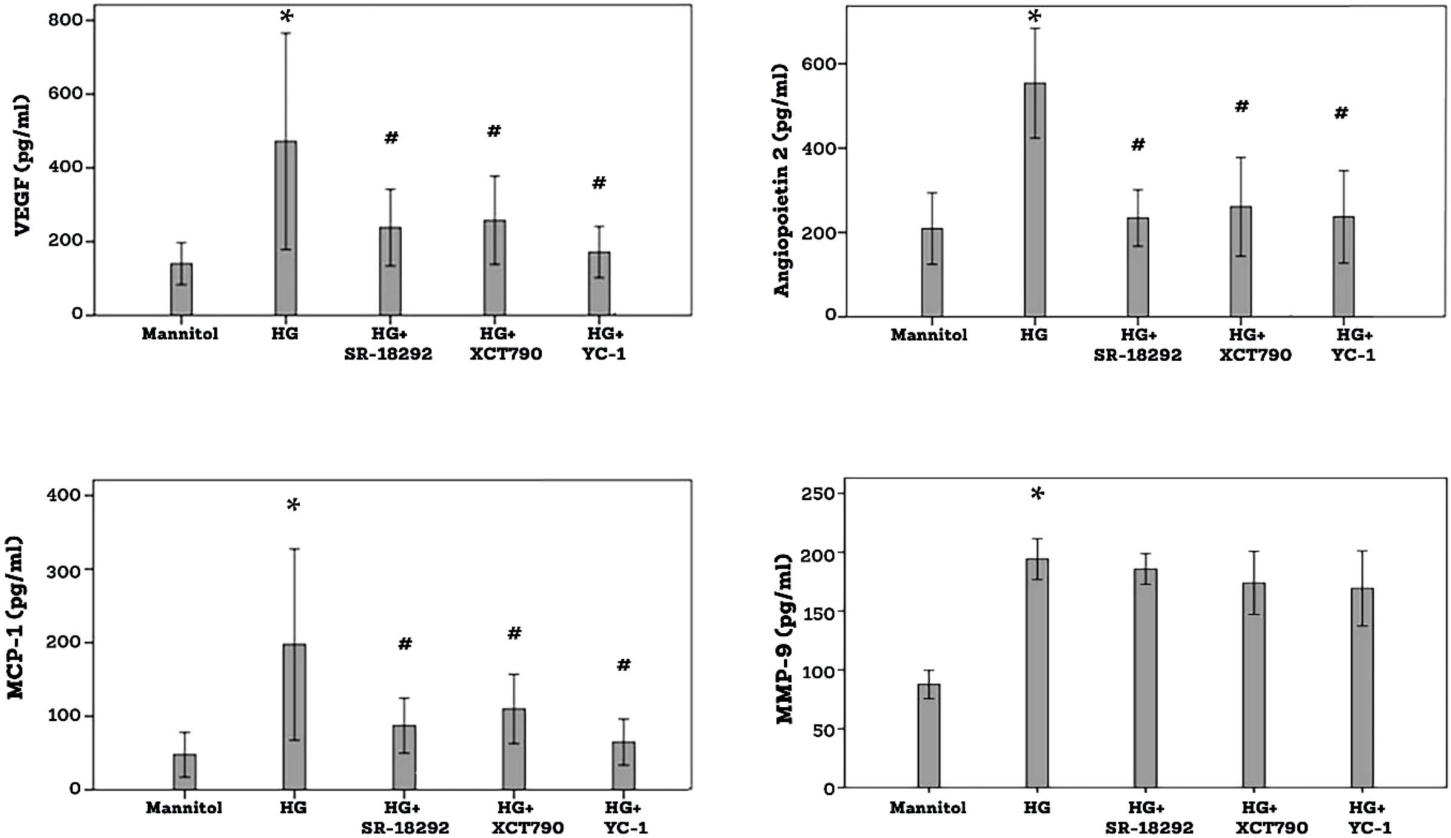
Human retinal Müller glial cells were left untreated or treated with high-glucose (HG) (25 mM) for 24 h or SR-18292 (20 µM), XCT790 (10 µM) or YC-1 (10 µM) for 1 h followed by HG. Cell cultures containing mannitol (25 mM) were used as a control. Levels of vascular endothelial growth factor (VEGF), angiopoietin 2, monocyte chemotactic protein-1 (MCP-1) and matrix metalloproteinase-9 (MMP-9) were quantified in the culture media by ELISA. Results are expressed as mean ± standard deviation from three different experiments, each performed in triplicate. One-way ANOVA and independent t-test were used for comparisons between five and two groups, respectively. P*< 0.05 compared with values obtained from mannitol-treated cells. p# <0.05 compared with values obtained from cells treated with HG.

ELISA analysis revealed that treatment of Müller cells with the diabetic mimetic condition high-glucose (HG) induced significant upregulation of the proangiogenic factors VEGF, angiopoietin 2 and MMP-9 and the inflammatory chemokine MCP-1/CCL2 in the culture medium as compared to untreated control. On the one hand, pretreatment with SR-1829, XCT790 or the HIF-1α transcription factor inhibitor YC-1 significantly attenuated the levels of VEGF, angiopoietin 2 and MCP-1/CCL2 induced by HG. On the other hand, SR-18292, XCT790 or YC-1 did not affect the expression of MMP-9 induced by HG (**Figure 9**).

### 3.8 Effect of the PGC-1α inhibitor SR-18292 and the ERR-α inhibitor XCT790 on CoCl_2_-induced upregulation of proangiogenic and inflammatory molecules in human retinal Müller glial cells

**Figure 10:**
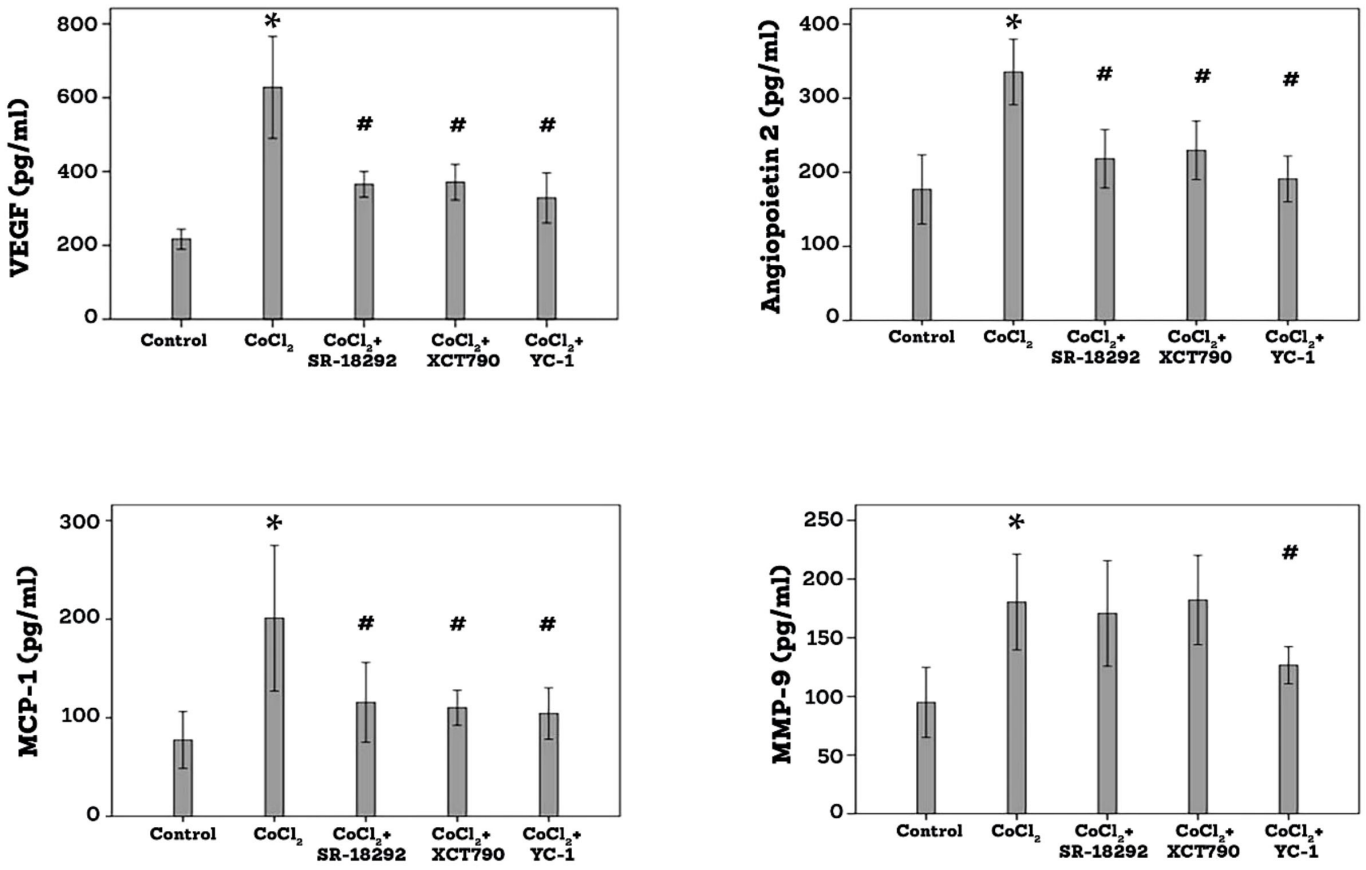
Human retinal Müller glial cells were left untreated or treated with cobalt chloride (CoCl_2_) (300µM) for 24 h or SR-18292 (20µM), XCT790 (10mM) or YC-1 (10µM) for 1 h followed by CoCl_2_. Levels of vascular endothelial growth factor (VEGF), angiopoietin 2, monocyte chemotactic protein-1 (MCP-1) and matrix metalloproteinase-9 (MMP-9) were quantified in the culture media by ELISA. Results are expressed as mean ± standard deviation from three different experiments, each performed in triplicate. One-way ANOVA and independent t-test were used for comparisons between five and two groups, respectively. *p<0.05 compared with values obtained from control cells. #p<0.005 compared with values obtained from cells treated with CoCl_2_.

ELISA analysis demonstrated that treatment of Müller cells with the hypoxia mimetic agent CoCl_2_ induced significant upregulation of VEGF, angiopoietin 2, MMP-9 and MCP-1/CCL2 in the culture medium as compared to untreated control. Pretreatment of Müller cells with SR-18292, XCT-90 or YC-1 significantly attenuated the levels of VEGF, angiopoietin 2 and MCP-1/CCL2. SR-18292 and XCT790 did not affect the expression of MMP-9, however, YC-1 significantly attenuated the levels of MMP-9 induced by CoCl_2_ (**Figure 10**).

Collectively, these data indicated that the PGC-1α/ERR-α pathway is required for the diabetic mimetic conditions-induced upregulation of VEGF, angiopoietin 2 and MCP-1/CCL2.

### 3.9 Effect of the PGC-1α transcriptional activator ZLN005 on the expression of proangiogenic and inflammatory molecules in human retinal Müller glial cells

**Figure 11:**
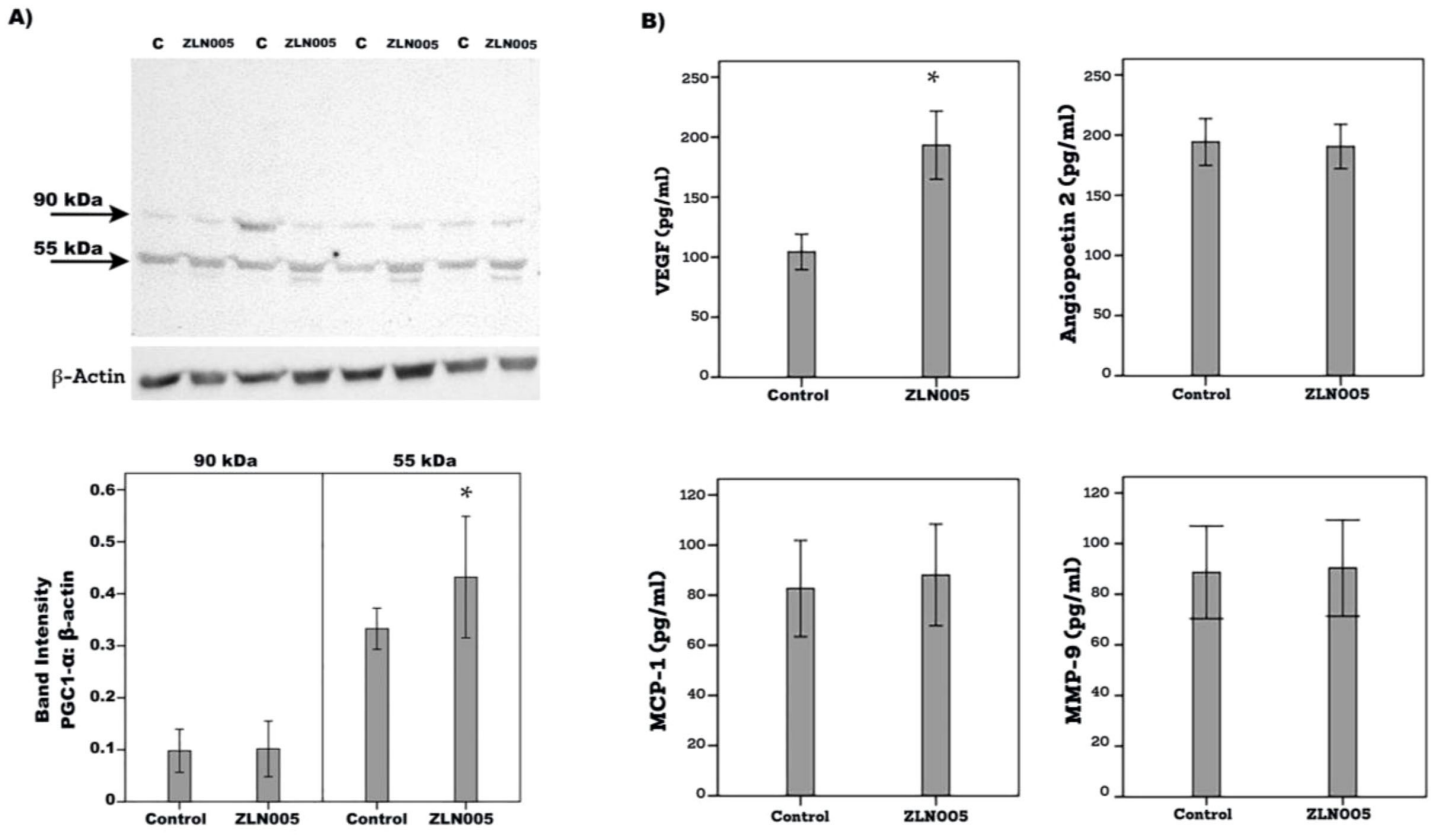
Human retinal Müller glial cells were left untreated (C) or treated with ZLN005 (20µM) for 48 h. The effect of ZLN005 on PGC-1α protein expression levels was determined with the use of Western blot analysis **(A)**. Levels of vascular endothelial growth factor (VEGF), angiopoietin 2, monocyte chemotactic protein-1 (MCP-1) and matrix metalloproteinase-9 (MMP-9) were quantified in the culture media by ELISA **(B)**. The results are expressed as mean ± standard deviation from three different experiments performed in triplicate. (p* < 0.05; independent t-test).

With the use of Western blot analysis, we demonstrated that treatment of Müller cells with ZLN005 for 48 h induced significant upregulation of PGC-1α as compared to untreated control (**Figure 11A**). With the use of ELISA analysis, we demonstrated that treatment of Müller cells with ZLN005 induced significant upregulation of the proangiogenic factor VEGF, but not angiopoietin 2, MMP-9 and MCP-1/CCL2, in the culture medium as compared to untreated control (**Figure 11B**). These findings suggest that ZLN005 increased PGC-1α and its downstream transcription factor in Müller cells.

### 3.10 Effect of the PGC-1α transcriptional activator ZLN005 on the generation of reactive oxygen species in human retinal Müller glial cells cultured in high-glucose

**Figure 12:**
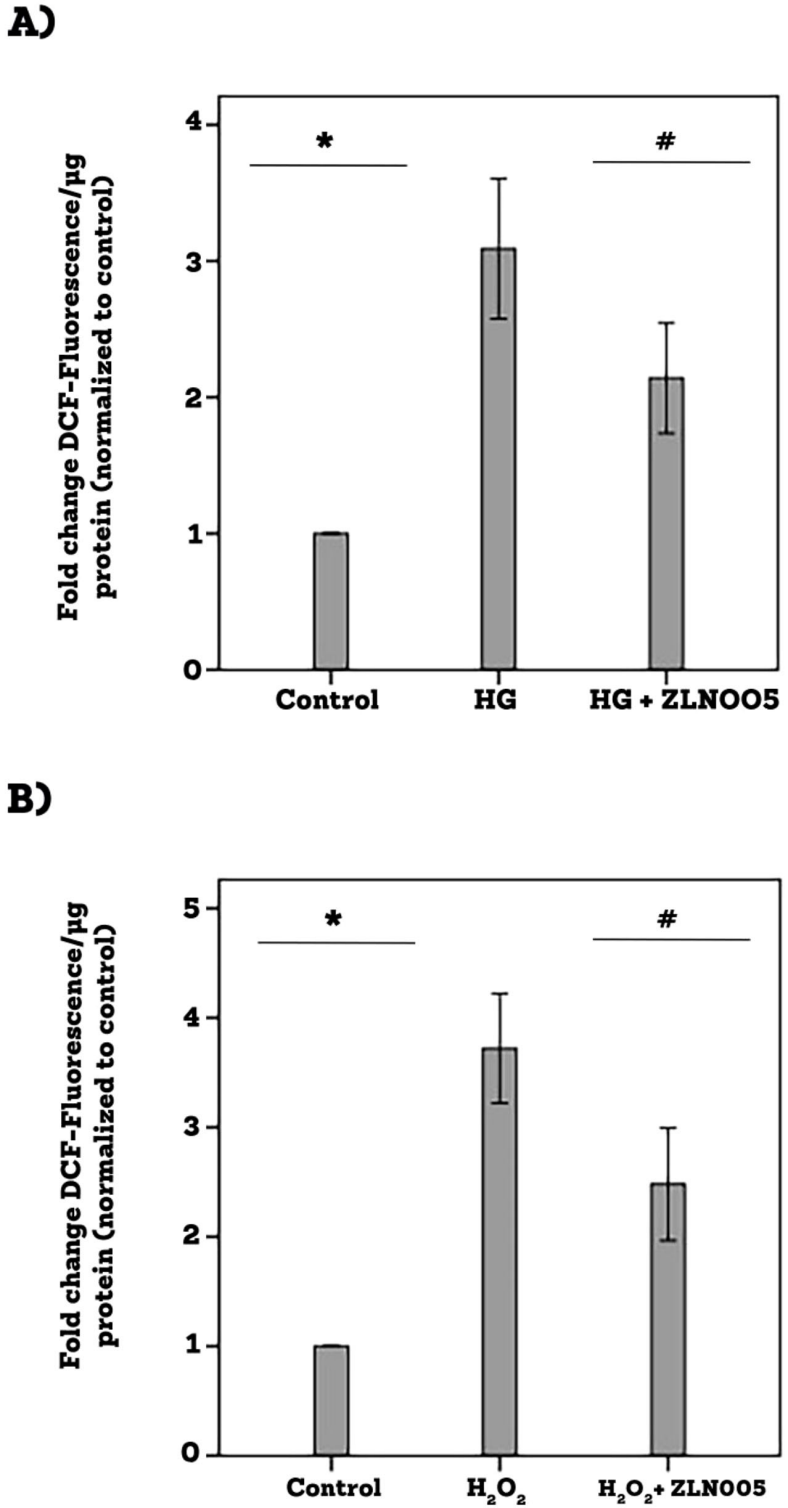
Human retinal Müller glial cells were left untreated or treated with high-glucose (HG) (25 mM) for 24 h **(A)** or hydrogen peroxide (H_2_O_2_) (10 µM) for 1 h **(B)** or ZLN005 (20 µM) for 1 h followed by HG or H_2_O_2_. For HG treatment, cultures containing 25 mM mannitol were used as a control. Oxidative stress was monitored with the use of 2’-7’-dichlorofluorescein (DCF) fluorescence intensity analysis. Results are expressed as mean ± standard deviation from three different experiments, each performed in triplicate. One-way ANOVA and independent t-test were used for comparisons between three and two groups, respectively. *p<0.05 compared with values obtained from control cells. #p<0.05 compared with values obtained from cells treated with HG or H_2_O_2_.

**Figure 12** demonstrates the changes in the signal of DCF fluorescence, which is an indicator of reactive oxygen species (ROS), in Müller cells. High-glucose induced significant upregulation of DCF fluorescence as compared to untreated control. Pretreatment with ZLN005 significantly attenuated ROS generation induced by high-glucose (**Figure 12A**). Treatment of Müller cells with exogenous reactive oxygen species H_2_O_2_ induced ROS generation. Pretreatment with ZLN005 significantly downregulated H_2_O_2_-induced ROS generation (**Figure 12B**).

## 4 Discussion

In this study, we demonstrated that PGC-1α and ERR-α levels were significantly upregulated in vitreous fluid samples from patients with PDR and were expressed in epiretinal fibrovascular membranes from patients with PDR. Immunohistochemical analysis demonstrated co-expression of PGC-1α and ERR-α proteins in endothelial cells lining pathologic new blood vessels and in leukocytes in PDR epiretinal fibrovascular membranes. In addition, angiogenic activity (CD31-positive vessels) correlated significantly with PGC-1α and ERR-α expressions in PDR fibrovascular membranes. We demonstrated that HRMECs constitutively express PGC-1α and ERR-α. Treatment of HRMECs with high-glucose or the hypoxia-mimetic agent CoCl_2_ did not affect the expression of PGC-1α or ERR-α. One possible explanation is the fact that this *in vitro* model represents short-term effects of high-glucose and hypoxia (24h), whereas in the patients, the disease evolved over much longer time intervals, eventually years. Additional positive correlations were demonstrated between vitreous fluid levels of PGC-1α and angiopoietin 2 and between the angiogenic biomarkers VEGF and angiopoietin 2. Consistent with our results in clinical samples, we demonstrated that PGC-1α and ERR-α protein levels were upregulated in the retina of streptozotocin-induced diabetic rats. Co-expression of PGC1-α and ERR-α strongly drives VEGF transcription (12). Although regulation of VEGF through HIF-1α is considered the classical pathway under ischemic conditions, a pathway involving PGC-1α and ERR-α was found to be independent of HIF-1α (11–13). Our results collectively suggest that upregulation of PGC-1α and ERR-α in the ocular microenvironment of patients with PDR is involved in angiogenic factors induction and in the initiation and progression of PDR.

There are several isoforms of PGC-1α protein, which arise by alternative splicing and are regulated possibly by specific promoter activities (23). The major proteoforms of PGC-1α are suggested to differ depending on the species and type of tissues, or experimental condition (22). Interestingly, in the present study, we demonstrated that the predominant PGC-1α proteoform in vitreous samples was found at 40 kDa. This band is suggested to represent the N-terminal part of the longer PGC-1α expressed proteoforms (20–22). It was demonstrated that hypoxic insults specifically induce alternatively spliced species encoding for truncated isoforms of PGC-1α in skeletal muscle cells leading to promotion of VEGF expression and secretion (21). The aminoterminal proteoform of PGC-1α, as compared with the full-length-PGC-1α preferentially induces an angiogenic program, whereas having a little effect on mitochondrial biogenesis in skeletal muscle cells. It was proposed that the specificity of the aminoterminal part of PGC-1α for the angiogenic program is achieved via specific binding to ERR-α (21). This suggests a mechanism by which the PGC-1α/ERR-α pathway can mediate hypoxic response without affecting mitochondrial genes.

Accumulating evidence suggests that the PGC-1α/ERR-α axis plays a vital role in the development and progression of several types of cancer and its overexpression is associated with poor prognosis. Moreover, the PGC-1α/ERR-α axis efficiently induces VEGF in cancer cells and promotes angiogenesis (12, 24–29). In addition, previous studies demonstrated a critical role of PGC-1α in the pathogenesis of pathological retinal neo-vascularization in the mouse model of oxygen-induced retinopathy. Suppression of PGC-1α expression resulted in reduction of VEGF expression and inhibited pathological retinal neovascularization (30, 31).

We dissected the role of the PGC-1α/ERR-α pathway in the pathogenesis of PDR at the cellular level *in vitro*. Retinal Müller glial cells are a major source of VEGF and other angiogenic factors and contribute to the development of pathological retinal neovascularization (32). We demonstrated that Müller cells constitutively express PGC-1α and ERR-α. In this study, we used the PGC-1α Specific inhibitor SR-18292 (33) and the ERR-α selective inverse agonist XCT790 (34, 35) as tools and probes to investigate the PGC-1α/ERR-α pathway functionally. We demonstrated that pretreatment of Müller cells with the PGC-1α inhibitor SR-18292 or the ERR-α antagonist XCT790 significantly attenuated the levels of the angiogenic factors VEGF and angiopoietin 2 and the inflammatory chemokine MCP-1/CCL2 induced by high-glucose or CoCl_2_. These results are consistent with a previous report showing that human retinal Müller glial cells express PGC-1α, that exposure of Müller cells to hypoxia enhanced VEGF expression and secretion and that inhibition of PGC-1α suppressed hypoxia-induced VEGF expression (30). These results are also consistent with a previous study, demonstrating that XCT790 treatment significantly decreased the production of VEGF and proliferation, migration and tube formation of human umbilical vein endothelial cells (36). In the animal model of spinal cord injury, XCT790 injection reduced the expression of VEGF and angiopoietin 2 and decreased vascular density and endothelial cell proliferation (37). In addition, XCT790 inhibited VEGF transcriptional activity, proliferation of tumor cells and inhibited *in vivo* tumor growth and angiogenesis (29, 38, 39). We also demonstrated that the HIF-1α transcription factor synthetic inhibitor YC-1 (40) had a similar inhibitory effect on the expression of VEGF, angiopoietin 2 and MCP-1/CCL2 induced by HG or CoCl_2_. Additionally, YC-1 attenuated upregulation of MMP-9 induced by CoCl_2_, but not by HG.

In addition, we demonstrated that treatment of Müller cells with ZLN005, a PGC-1α transcriptional activator that upregulates PGC-1α expression (41, 42), induced significant upregulation of VEGF, and attenuated production of ROS by Müller cells following exposure to HG and H_2_0_2_- induced oxidative stress. Similar findings were previously reported (42, 43). A previous study demonstrated that PGC-1α control of ROS homeostasis plays an important role in the control of endothelial response to VEGF and angiogenesis. Elevated production of ROS in the absence of PGC-1α was found to be a key factor in the alteration of the VEGF signaling pathway and the capacity of endothelial cells to form stable interactions with other endothelial cells and with the extracellular matrix (44).

In conclusion, in the present study we demonstrated for the first time that the components of the PGC-1α/ERR-α pathway are significantly upregulated in the intraocular microenvironment of patients with PDR and in a rat model of acute inflammation induced by diabetes. Suppression of PGC-1α and ERR-α impairs the upregulation of angiogenic and inflammatory factors by Müller glial cells induced by the studied diabetic mimetic conditions. These findings suggest that the PGC-1α/ERR-α pathway is an angiogenic pathway with clinical relevance in patients with PDR. Targeting the PGC-1α/ERR-α pathway may improve the anti-angiogenic effect of agents in clinical use.

## Data Availability

The raw data supporting the conclusions of this article will be made available by the authors, without undue reservation.
